# Neuroprotective Effects of Sevoflurane against Electromagnetic Pulse-Induced Brain Injury through Inhibition of Neuronal Oxidative Stress and Apoptosis

**DOI:** 10.1371/journal.pone.0091019

**Published:** 2014-03-10

**Authors:** Bin Deng, Hao Xu, Jin Zhang, Jin Wang, Li-Chun Han, Li-Ya Li, Guang-Li Wu, Yan-Ning Hou, Guo-Zhen Guo, Qiang Wang, Han-Fei Sang, Li-Xian Xu

**Affiliations:** 1 State Key Laboratory of Military Stomatology, Department of Anesthesiology, School of Stomatology, The Fourth Military Medical University, Xi'an, China; 2 Department of Anatomy, Histology and Embryology, K. K. Leung Brain Research Centre, the Fourth Military Medical University, Xi'an, China; 3 Department of Clinical Pharmacology, Bethune International Peace Hospital of People's Liberation Army, Shijiazhuang, China; 4 Department of Radiation Medicine, Faculty of Preventive Medicine, the Fourth Military Medical University, Xi'an, China; 5 Department of Anesthesiology, Shannxi Tumor Hospital, Xi'an, China; 6 Department of Anesthesiology, Xijing Hospital, The Fourth Military Medical University, Xi'an, China; 7 Department of Nephrology, Bethune International Peace Hospital of People?'s Liberation Army, Shijiazhuang, China; Massachusetts General Hospital, United States of America

## Abstract

Electromagnetic pulse (EMP) causes central nervous system damage and neurobehavioral disorders, and sevoflurane protects the brain from ischemic injury. We investigated the effects of sevoflurane on EMP-induced brain injury. Rats were exposed to EMP and immediately treated with sevoflurane. The protective effects of sevoflurane were assessed by Nissl staining, Fluoro-Jade C staining and electron microscopy. The neurobehavioral effects were assessed using the open-field test and the Morris water maze. Finally, primary cerebral cortical neurons were exposed to EMP and incubated with different concentration of sevoflurane. The cellular viability, lactate dehydrogenase (LDH) release, superoxide dismutase (SOD) activity and malondialdehyde (MDA) level were assayed. TUNEL staining was performed, and the expression of apoptotic markers was determined. The cerebral cortexes of EMP-exposed rats presented neuronal abnormalities. Sevoflurane alleviated these effects, as well as the learning and memory deficits caused by EMP exposure. In vitro, cell viability was reduced and LDH release was increased after EMP exposure; treatment with sevoflurane ameliorated these effects. Additionally, sevoflurane increased SOD activity, decreased MDA levels and alleviated neuronal apoptosis by regulating the expression of cleaved caspase-3, Bax and Bcl-2. These findings demonstrate that Sevoflurane conferred neuroprotective effects against EMP radiation-induced brain damage by inhibiting neuronal oxidative stress and apoptosis.

## Introduction

Most electrical equipment and wireless communication devices produce electromagnetic radiation. There is widespread concern regarding the adverse effects on human health caused by exposure to many types of electromagnetic fields (EMFs) [1], [2]. The potential for EMF exposure to damage the central nervous system (CNS) has been discussed in-depth. Previous studies indicate that the non-thermal effects of EMF exposure can lead to cellular changes [3], [4], [5]. Additionally, EMF can increase reactive oxygen species (ROS) and reactive nitrogen species (RNS) in organs and cause histopathological damage and oxidative stress [6], [7], [8], [9]; for example, under particular circumstances, exposure to a GSM-modulated, 900-MHz signal acts as a co-stressor for oxidative damage of neural cells [10]. Several studies also suggest that occupational exposure to electromagnetic fields may be associated with increased risk of neurodegenerative diseases [11], [12].

Electromagnetic pulse (EMP), a specific type of EMF, is a short high-voltage pulse with an extremely fast rising time and a broad bandwidth from extremely low frequencies up to 1.5 GHz [13], [14]. EMP is widely applied in medical therapies, such as those targeting osteoporosis, and is also used in military campaigns. However, the biological effects and potential harm to humans in an environment of electromagnetic radiation have not been well studied. Brain tissue is sensitive to EMP, which increases cerebral microvascular permeability in rats [15] and can disrupt the blood-brain barrier (BBB) [5]. Additionally, EMP exposure can cause long-term impairments in rat learning and memory [12]. However, the non-thermal effects of EMP remain controversial [2]. It is unknown whether the non-thermal effects of EMP can induce short-term histopathological damage and ultrastructural changes in cerebral cortex neurons.

Recently, the neuroprotective effect of inhaled anesthetics has attracted increased attention. Sevoflurane is an inhaled anesthetic that is broadly used in clinical applications [16], [17], . The neuroprotective effect of sevoflurane may be related to its inhibitory effects on oxidative stress, apoptosis and excitatory amino acid release and its stabilizing effects on cell metabolism [19], [20], [21], [22]. However, it is unclear whether sevoflurane exerts neuroprotective effects against EMP radiation-induced brain injury.

The present study was designed to investigate whether exposure to EMP (400 kV/m, 200 pulses) has detrimental effects on cerebral cortex neurons and cognitive ability and to further elucidate whether sevoflurane exerts neuroprotective effects against EMP radiation-induced cerebral damage *in vivo* and *in vitro*.

## Materials and Methods

### 1. Experimental protocols

#### Experiment 1

To assess the effect of EMP on cerebral cortex neurons, male Sprague–Dawley (SD) rats were randomly divided into 4 groups (*n* = 6 each): control (sham exposure) group, 1 h after EMP group, 6 h after EMP group and 24 h after EMP group. The animals were exposed or not to 200 pulses of EMP at 400 kV/m. At 1, 6 and 24 h after EMP exposure, HE staining was performed to observe the effects of EMP on cerebral cortex neurons.

#### Experiment 2

To determine whether sevoflurane treatment alleviates EMP-induced injury, SD rats were randomly divided into 4 groups (*n* = 6 each): EMP group, 1% sevoflurane treatment group, 2% sevoflurane treatment group and 4% sevoflurane treatment group. The rats were exposed or not to 200 pulses of EMP at 400 kV/m. After EMP exposure, the rats were immediately treated with 1, 2 or 4% sevoflurane for 20 min. At 24 h after EMP exposure, the protective effects of sevoflurane were assessed by Nissl staining, Fluoro-Jade C (FJC) staining and electron microscopy. Meanwhile, additional SD rats were randomly divided into 3 groups (*n* = 6 each): control (sham exposure) group, EMP group and 2% sevoflurane treatment group. At 24 h after EMP exposure, the protective effects of sevoflurane were assessed by neurobehavioral testing.

#### Experiment 3

To further explore the neuroprotective effect of sevoflurane against EMP-induced injury and the underlying mechanism, cultured primary cerebral cortical neurons were randomly divided into 5 groups: control (sham exposure) group, EMP group, 1% sevoflurane treatment group, 2% sevoflurane treatment group and 4% sevoflurane treatment group. The cells were exposed or not to 200 pulses of EMP at 400 kV/m. After EMP exposure, the cells were immediately incubated with 1, 2 or 4% sevoflurane for 20 min. Cell viability, LDH release, SOD activity and MDA levels at 24 h after EMP exposure were measured. Additionally, TUNEL staining and western blotting of cleaved caspase-3, Bcl-2, and Bax were performed at 24 h after EMP exposure.

### 2. Animals

Male SD rats weighing 200–220 g and SD rat embryos (E18) were obtained from the Animal Center of the Fourth Military Medical University (Xi'an, China). All animals received only one single exposure/sham exposure. The animals were housed in a specific pathogen-free environment with free access to sterile laboratory pellets and water, and they were fed without further contact with EMP. The experimental protocol was approved by the Ethics Committee for Animal Experimentation of the Fourth Military Medical University and was conducted according to the Guidelines for Animal Experimentation of the Fourth Military Medical University (Xi'an, China).

### 3. EMP exposure

**Figure 1:**
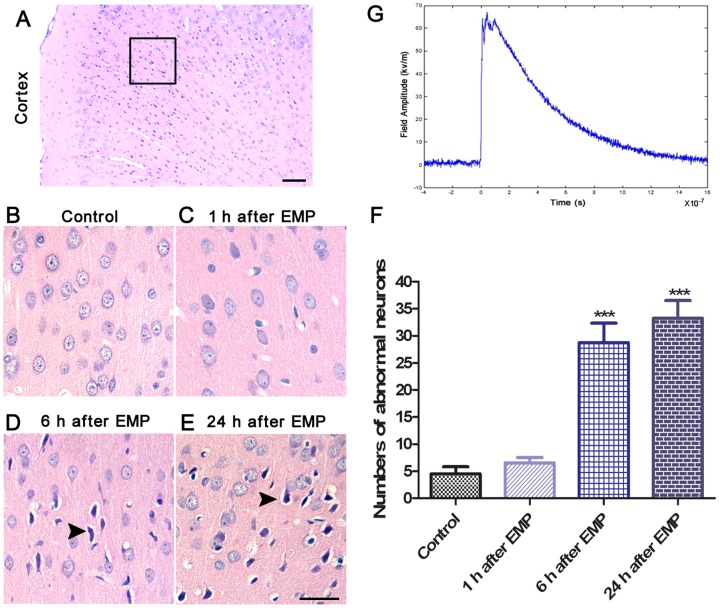
Histological characteristics of cerebral cortex neurons in rats exposed to EMP radiation (n = 6). **A**, The highlighted box is the observation region (scale bar = 50 µm). **B–E**, Representative HE staining of cerebral cortex neurons in control rats or rats at 1, 6 and 24 h after exposure to EMP radiation. Damaged neurons are characterized by a darkly stained nucleus and cytoplasm (arrow) (scale bar = 50 µm). **F**, The number of abnormal neurons in the cerebral cortex. The data are presented as the mean±SEM. ***P<0.01 vs. control group. **G,** Waveform of EMP. The EMP (peak-intensity 400 kV/m, rise-time 10 ns, pulse width 350 ns, 200 pulses) was generated by a spark gap pulse generator and transmitted into a Gigahertz transverse electromagnetic cell.

An all-solid-state nanosecond generator was developed and tested as described in previous studies [5], [13]. Briefly, EMP (peak intensity 400 KV/m, rise time 10 ns, pulse width 350 ns, 0.5 pps, 1 Hz, 200 pulses total) was generated by a spark gap generator and transmitted into a gigahertz transverse electromagnetic (GTEM) cell. The electric field in the exposure area was uniform within 30×30×30 cm. The animals were whole-body exposed to EMP at 400 kV/m for 200 pulses (Fig. 1G). During exposure, the rats were awake and not restrained in the exposure chamber. The temperature measurements were made immediately before and after EMP exposure. The exposure caused a <0.2°C increase in the rectal temperature of control and exposed rats. The cells were also exposed to EMP at 400 kV/m for 200 pulses. After exposure, no significant change in the temperature of the medium was noted. A Tektronix 7000B oscilloscope (Tektronix, Beaverton, OR) was used to observe and record the pulse waveform.

### 4. Sevoflurane treatment

After EMP exposure, the rats were exposed to 1, 2 or 4% sevoflurane in 100% oxygen for 20 min in a glass chamber. The gas pressure was continuously monitored. After 20 min, the rats were allowed to recover and were returned to their home cages for monitoring. At 24 h after EMP exposure, the rats were sacrificed, and brain sections were prepared for histology or immunocytochemical staining and electron microscopy.

After EMP exposure *in vitro*, the cells were exposed to 1, 2 or 4% sevoflurane in 100% oxygen for 20 min in a glass chamber. The gas pressure was continuously monitored. After 20 min, the cells were allowed to recover at 37°C in a humidified incubator under 95% air/5% CO_2_ and monitored. At 24 h after EMP exposure, the cells were used for further study.

### 5. Open-field test

At 24 h after EMP injury, rats from the control group, EMP group and 2% sevoflurane treatment group (*n* = 6 each) were subjected to an open-field test. The open-field apparatus consisted of a square area (90×90 cm) with opaque walls that were 45 cm high. The apparatus was placed in indirect light (60 Lux). The rats were placed in a corner of the open field, facing the opaque walls. The distance traversed in the central area and the duration of movement in the outer ring was then observed for 15 min on a monitor through a video camera system. After the removal of each animal, the apparatus was cleaned.

### 6. Water maze task

At 24 h after EMP injury, the rats were submitted to behavioral testing to analyze spatial learning and memory in the Morris water maze. The water maze was a circular pool (painted black, 1.8 m in diameter, 0.4 m high) constructed of fiberglass. The water was maintained at 22±2°C. The pool was geographically divided into four equal quadrants, with release points in each quadrant designated as southwest, northwest, northeast and southeast. During testing in the water maze, a transparent platform (8 cm in diameter) was placed 1 cm beneath the water surface. This escape platform location was kept constant in the middle of the southwest quadrant. Rats were placed into the water facing the wall at each release point in a random order. A video camera was mounted in the ceiling above the pool and was connected to a video recorder and tracking device (S-MART; Pan-Lab, Barcelona, Spain), which permitted online and offline automated tracking of the paths taken by the rats. The animals were subjected to four trials per session. The rats were trained to locate the hidden escape platform, which remained in a fixed location throughout the testing. Trials lasted a maximum of 120 s and the latency and the experimenter placed rats in platform for 15 sec. The intertrial interval was 60 sec. Each rat performed 4 trials daily for 4 days. During the training trials, the length of the path by which each animal found the platform was measured. On the fifth day, the rats were subjected to a 2-min probe trial in which the platform was removed. The start point for the probe trial was randomly selected for each subject. The swimming time and trajectory of the rats were recorded.

### 7. HE staining and Nissl staining

At 24 h after EMP exposure, the rats were perfused with 4% (w/v) paraformaldehyde. The brain tissues were removed, placed in 20% (w/v) sucrose and sectioned at 10 µm. The sections were processed for HE staining and Nissl staining [23]. For HE staining, the sections were deparaffinized with xylene and dehydrated. Hematoxylin staining was performed for 5 min, followed by washing in tap water with a 10-s rinse in HCl solution (0.1%). After an additional wash in tap water, the sections were stained in eosin solution for 2 min, washed in tap water, dehydrated and cleared. Finally, the sections were mounted in neutral gum. Nissl staining (Beyotime Institute of Biotechnology, China) was performed to detect Nissl bodies in the cytoplasm of surviving neurons. The integrated optical density/area of the staining in each group was acquired by 2 blinded investigators using ImagePro Plus 5.1 software (Media Cybernetics, Inc., Bethesda, MD).

### 8. FJC staining

FJC (50 mg) was purchased from Chemicon (catalog number: AG325, Temecula, CA 92590, USA). The preparation of brain tissue sections (16 µm thickness) for fluorescence microscopy and the FJC staining were performed as described previously [24] and according to manufacturer's instructions. The slides were observed and photographed under a fluorescence microscope as described above.

### 9. Electron microscopy analysis

The animals were anaesthetized intraperitoneally with 1% sodium pentobarbital (50 mg/kg body weight) and perfused transcardially with 150 ml of warm saline followed by 500 ml of ice-cold fixative for 30 min. A mixture of 1% paraformaldehyde and 2% glutaraldehyde in 0.1 M sodium phosphate buffer (pH 7.4) was used as a fixative. A segment of the brain was then removed and postfixed by immersion in the same fixative for 2 h at 4°C. We then prepared ultrathin sections following standard procedures and observed the sections using a JEM-100SX electron microscope.

### 10. Primary cerebral cortical neuron culture

Primary cerebral cortical neurons were cultured as described previously [25]. Briefly, the neurons were isolated from rat embryos (E18), washed three times with D-Hanks' solution under sterile conditions and subsequently seeded at a density of 1×10^6^ cells/cm^2^ onto plates coated with poly-L-lysine (50 µg/ml) (Sigma-Aldrich Co, USA). These cells were grown in neurobasal medium (Gibco, Invitrogen Corp, USA) supplemented with 2% B27, 1% glutamine and 1% penicillin/streptomycin (Sigma-Aldrich Corp, USA) at 37°C in a humidified incubator under 95% air/5% CO_2_. The purity of the neurons was determined by immunocytochemistry for βIII-tubulin (1∶250; Millipore, Temecula, CA, USA), which indicated that 95% of the cells in the cultures were βIII-tubulin positive (data not shown).

### 11. Methyl-thiazolyl-tetrazolium (MTT) assay and measurement of LDH release

At 24 h after EMP exposure or sevoflurane treatment (Experiment 3), cell viability was assessed by the MTT assay as previously described [26]. We also assessed lactate dehydrogenase (LDH) release, an indicator of neuronal injury, using a commercially available kit [25]. Briefly, cell-free culture supernatants (10 µl) were collected from each well and incubated with the appropriate reagent mixture at 37°C for 30 min according to the manufacturer's instructions (Nanjing Jiancheng, China). The reaction was terminated by adding five volumes of 0.1 M NaOH, and the absorbance was measured at 440 nm with an ELISA reader (Epoch™, BioTek, USA). The data were normalized to the control group and expressed as a percentage.

### 12. SOD activity and MDA level

The SOD activity and MDA level were measured using assay kits (A001 and A003; Nanjing Jiancheng Bioengineering Institute, Nanjing, China) following the manufacturer's instructions [27].

### 13. TUNEL staining

To determine whether sevoflurane can reduce apoptosis induced by EMP exposure, TUNEL staining was performed *in vivo* using an In Situ Cell Death Detection Kit (Roche Diagnostics, Mannheim, Germany) as described previously and according to the manufacturer's instructions [28]. In brief, neurons in every group were fixed with 4% (v/v) ice-cold paraformaldehyde for 1 h, washed in PBS (0.1 M, pH 7.4) for 5 min, treated with 0.3% (v/v) H_2_O_2_ for 10 min, rinsed in PBS for 5 min, incubated in a TUNEL reaction mixture for 1 h at 37°C and stained with DAPI for 5 min at room temperature. Images were obtained with a microscope (BX60, Olympus). The integrated optical density/area of the positive TUNEL staining in each group was acquired as described above.

### 14. Western blot

We analyzed the expression of active caspase 3, Bcl-2 and Bax by western blotting as described previously [29]. The total protein concentration of the cells was analyzed with a BCA kit (Sigma, CA, USA). The blots were probed with a rabbit antibody against cleaved (active) caspase-3 (17 kDa) (1∶1000; Cell Signaling Technology, Beverly, MA) and with mouse monoclonal antibodies against Bcl-2 or Bax (1∶1000, Santa Cruz, CA, USA) and β-actin (1∶2000; Anbo, Francisco, CA, USA). Subsequently, the blots were probed with a horseradish peroxidase (HRP)-conjugated goat secondary antibody against rabbit or mouse IgG (1∶1000; Abcam). Detection and quantitation were performed with a Typhoon 9400 Variable Mode Imager (GE Healthcare) and Lumi-Light Western Blotting Substrate (Roche Diagnostics) for HRP-labeled blots.

### 15. Statistical analysis

SPSS 13.0 for Windows (SPSS Inc., Chicago, IL) was used for statistical analyses. The neurological deficit scores (such as TMSs) were expressed as the median (range) and were analyzed with the Kruskal-Wallis test followed by the Mann-Whitney U statistic with Bonferroni correction. Other values were reported as the mean ± SEM and were analyzed among groups by one-way analysis of variance. When indicated by a significant F ratio, post hoc testing was performed with Scheffe's test. The statistical significance was set at *P*<0.05.

## Results

### 1. EMP radiation caused neuronal damage in rats

HE staining revealed that at 6 or 24 h after EMP radiation, the arrangement of abnormal neurons was irregular, and these cell bodies exhibited pyknosis, which is characterized by a darkly stained nucleus and cytoplasm. The number of abnormal neurons was significantly increased at 6 or 24 h after EMP radiation compared with sham EMP (Fig. 1). However, there was no significant difference between the EMP group and control group 1 h after treatment.

### 2. Sevoflurane alleviated neuronal damage and degeneration in rats exposed to EMP radiation

**Figure 2:**
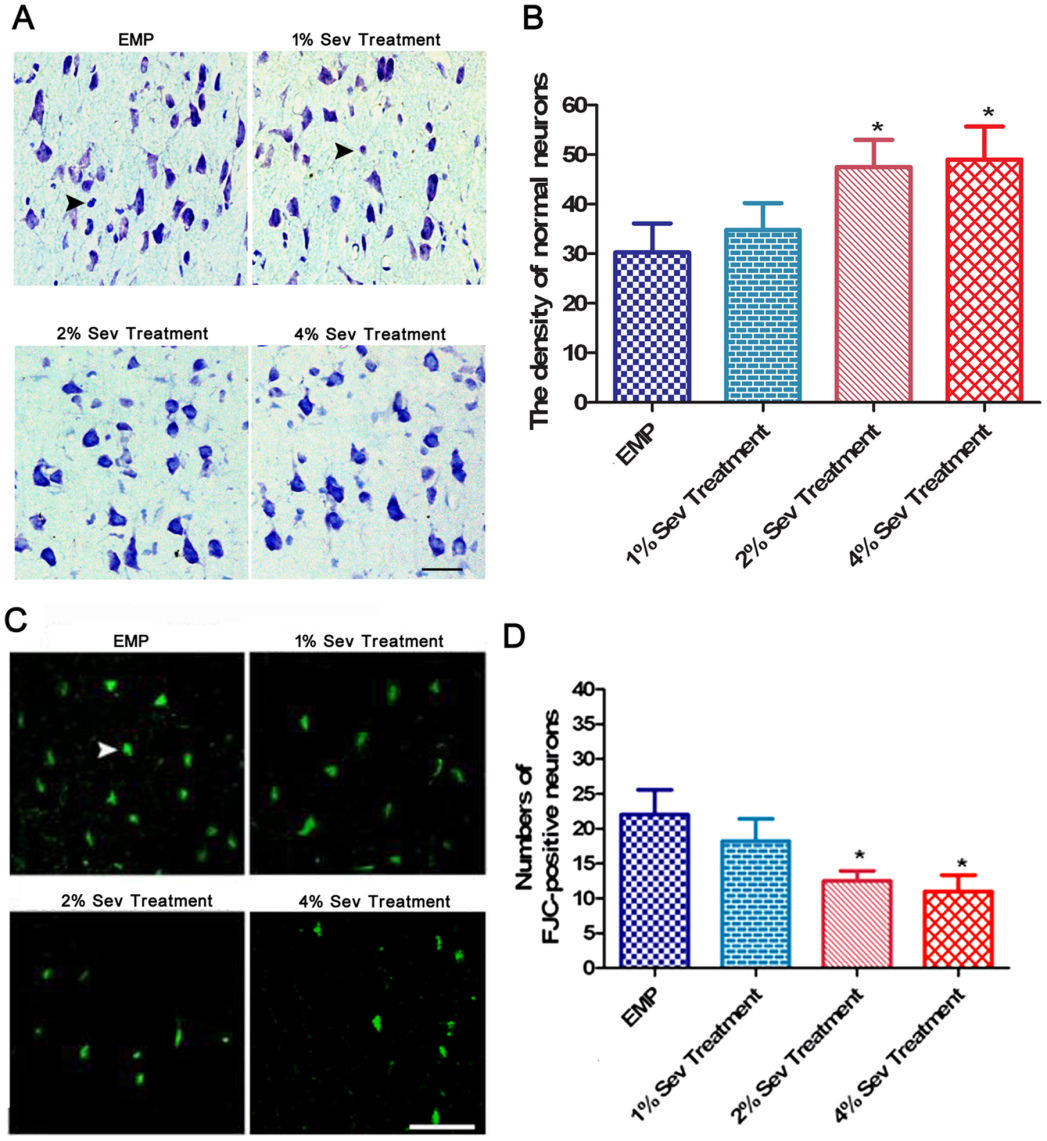
Effect of sevoflurane on neuronal morphology and degeneration of the cerebral cortex in rats exposed to EMP radiation (n = 6). **A**, Representative Nissl staining of cerebral cortex neurons in rats at 24 h after exposure to EMP radiation. The arrows indicate the morphology of abnormal neurons (scale bar = 50 µm). **B**, The density of normal neurons in the cerebral cortex at 24 h after EMP radiation. **C**, Representative FJC staining of cerebral cortex neurons in rats at 24 h after exposure to EMP radiation. Degenerative neurons were stained brightly by FJC. The arrows indicate the FJC-positive cells (scale bar = 50 µm). **D**, The number of FJC-positive neurons in the cerebral cortex at 24 h after EMP exposure. The data are presented as the mean±SEM. *P<0.05 vs. EMP group.

Nissl staining revealed neuronal injuries, including cell loss, cellular swelling, nuclear pyknosis and karyorrhexis, at 24 h after EMP exposure. Exposure to 2% and 4% sevoflurane alleviated the neuronal injury caused by EMP radiation (Fig. 2A). Compared with the EMP group, the density of normal neurons in the 2% and 4% sevoflurane treatment groups increased significantly (*P*<0.05) (Fig. 2B). However, there was no significant difference between the EMP group and the 1% sevoflurane treatment group.

FJC-positive cells exhibited neuronal processes with positive staining. The FJC-positive-stained degenerative cells exhibited shrunken or smaller cell bodies compared with normal neurons in the cerebral cortex region (Fig. 2C). Similarly, the number of FJC-positive neurons in the 2% and 4% sevoflurane groups was reduced significantly compared with that in the EMP group (*P*<0.05) at 24 h after EMP irradiation. However, there was no significant difference between the number of FJC-positive neurons in the 1% sevoflurane treatment group and the EMP group (Fig. 2D).

### 3. Sevoflurane protected neuronal ultrastructure in rats exposed to EMP radiation

**Figure 3:**
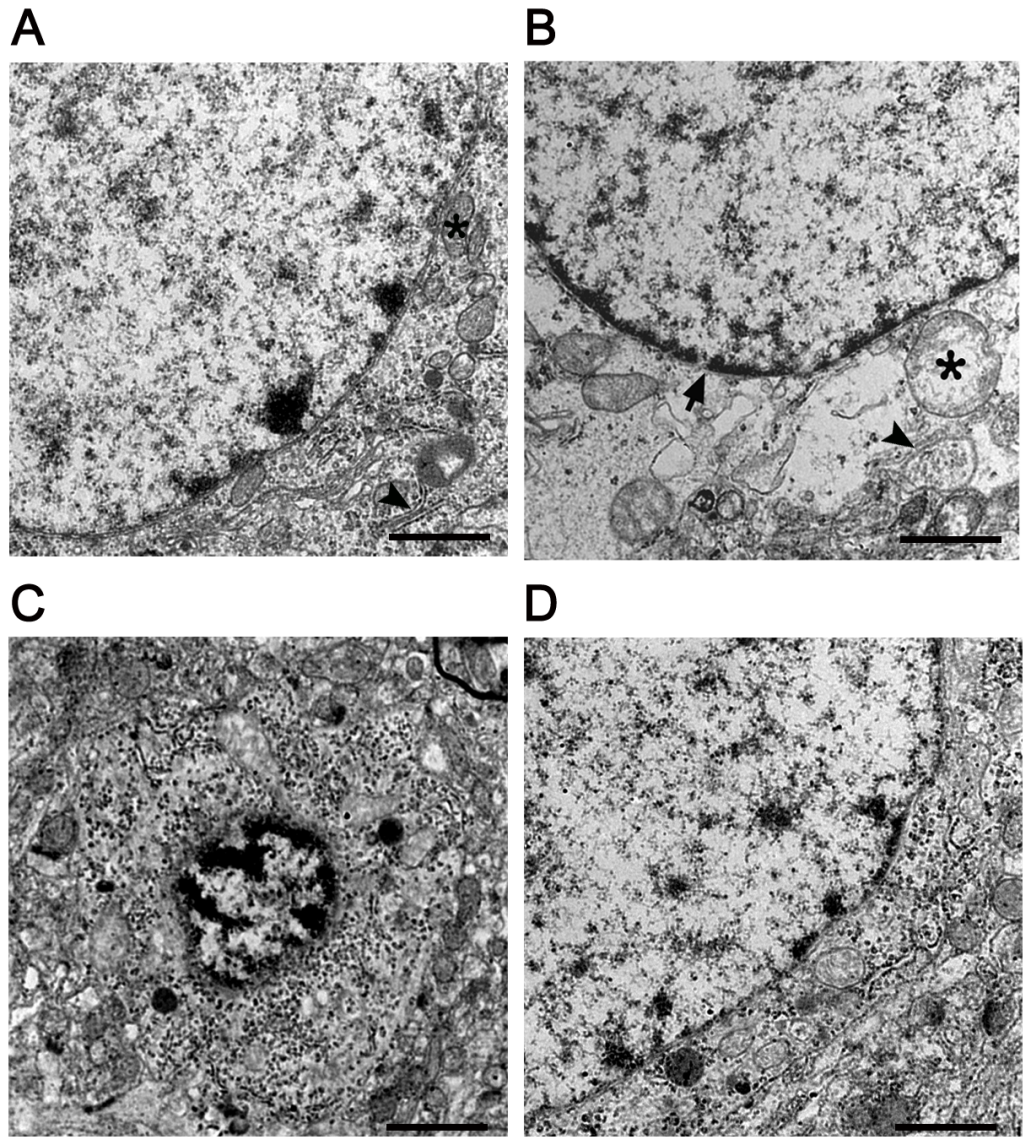
Effect of sevoflurane on the ultrastructure of the cerebral cortex neurons of rats at 24 h after EMP (n = 6). **A**, Electron microscopic image of normal cerebral cortex neurons. **B and C**, Electron microscopic image of cerebral cortex neurons at 24 h after EMP radiation. The shape of the neurons became obviously irregular. Mitochondria in the cytoplasm swelled to a spherical shape, the rough endoplasmic reticulum was cystic and the nuclear membrane was sunken, damaged and collapsed to varying degrees. D, Representative electron microscopic image of cerebral cortex neurons treated with 2% sevoflurane. Scale bar = 1 µm. * indicates: mitochondria in the cytoplasm; Triangular arrowheads indicates: endoplasmic reticulum; Arrow indicates nuclear membrane.

Electron micrographs of normal cerebral cortex neurons are shown in Fig. 3A. At 24 h after EMP exposure, the ultrastructure of cortical neurons was characterized by nuclear membrane folds, collapse, blurred boundaries, nuclear condensation and increased heterochromatin. A large amount of nuclear chromatin was accumulated under the nuclear membrane. Additionally, obvious swelling of mitochondria in the cytoplasm was noted, and many mitochondria were spherical in shape. Some mitochondrial cristae were fractured. The rough endoplasmic reticulum was cystic and degranulated. Moreover, the structure of the cerebral cortex was disrupted. The cytoplasm became more highly concentrated as the electron density increased, the nucleus shrank significantly, the chromatin was concentrated and aggregated at the edge of the nucleus and apoptotic bodies were observed that were similar to those observed in apoptotic cells (Figs. 3B and 3C). The damage to the neuronal ultrastructure in the 2% sevoflurane treatment group was significantly alleviated, especially in the mitochondria and the rough endoplasmic reticulum (Fig. 3D).

### 4. Sevoflurane improved cognitive ability in rats exposed to EMP radiation

**Figure 4:**
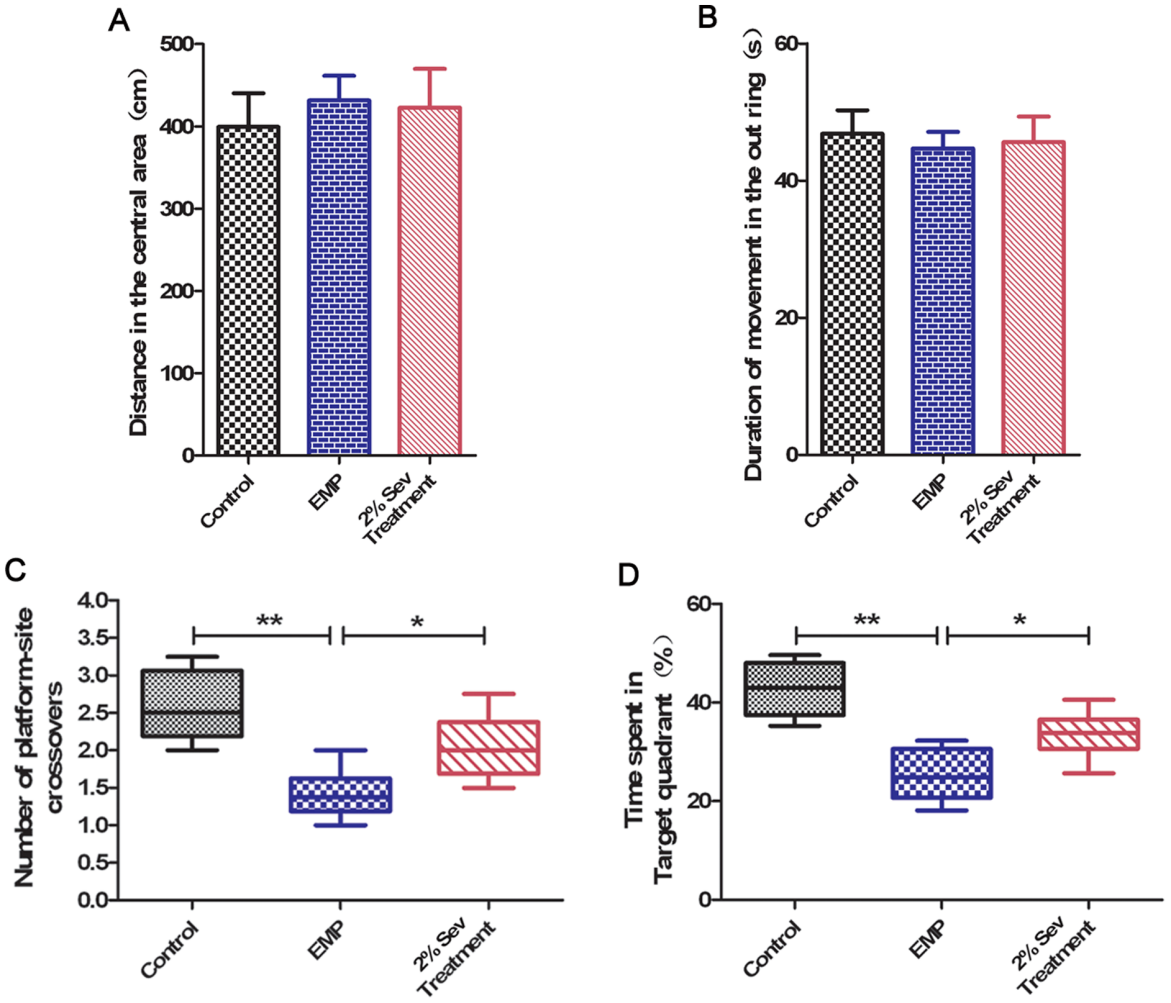
Effect of sevoflurane on the neurobehavioral ability of rats exposed to EMP radiation (n = 12). **A and B**, Motor function was assessed by a n open-field test at 24 h after EMP exposure. **C and D**, Learning and memory abilities were assessed by the Morris water maze and measured at 24 h after EMP exposure. *, *P*<0.05 and **, *P*<0.01.

Motor function recovery was evaluated by the open-field test. Rats in the 2% sevoflurane treatment group traversed a similar distance in the central area as those in the EMP group at 24 h after EMP injury (Fig. 4A). The duration of movement in the outer ring did not differ between the 2% sevoflurane treatment group and the EMP group at 24 h after EMP exposure (Fig. 4B).

Rats in the EMP group spent less time in the target quadrant (Fig. 4C) than rats in the control group (P<0.01); however, after 2% sevoflurane treatment, this time period was longer than that in the EMP group (*P*<0.05). The number of platform-site crossovers (Fig. 4D) in the EMP group was less than that in the control group (*P*<0.01); however, the number of crossovers was greater in the 2% sevoflurane treatment group compared with the EMP group (*P*<0.05). These results suggest that EMP radiation impaired the learning and memory of rats, whereas 2% sevoflurane treatment alleviated this learning and memory damage.

### 5. Sevoflurane attenuated the damage to cerebral cortical neurons induced by EMP radiation *in vitro*

**Figure 5:**
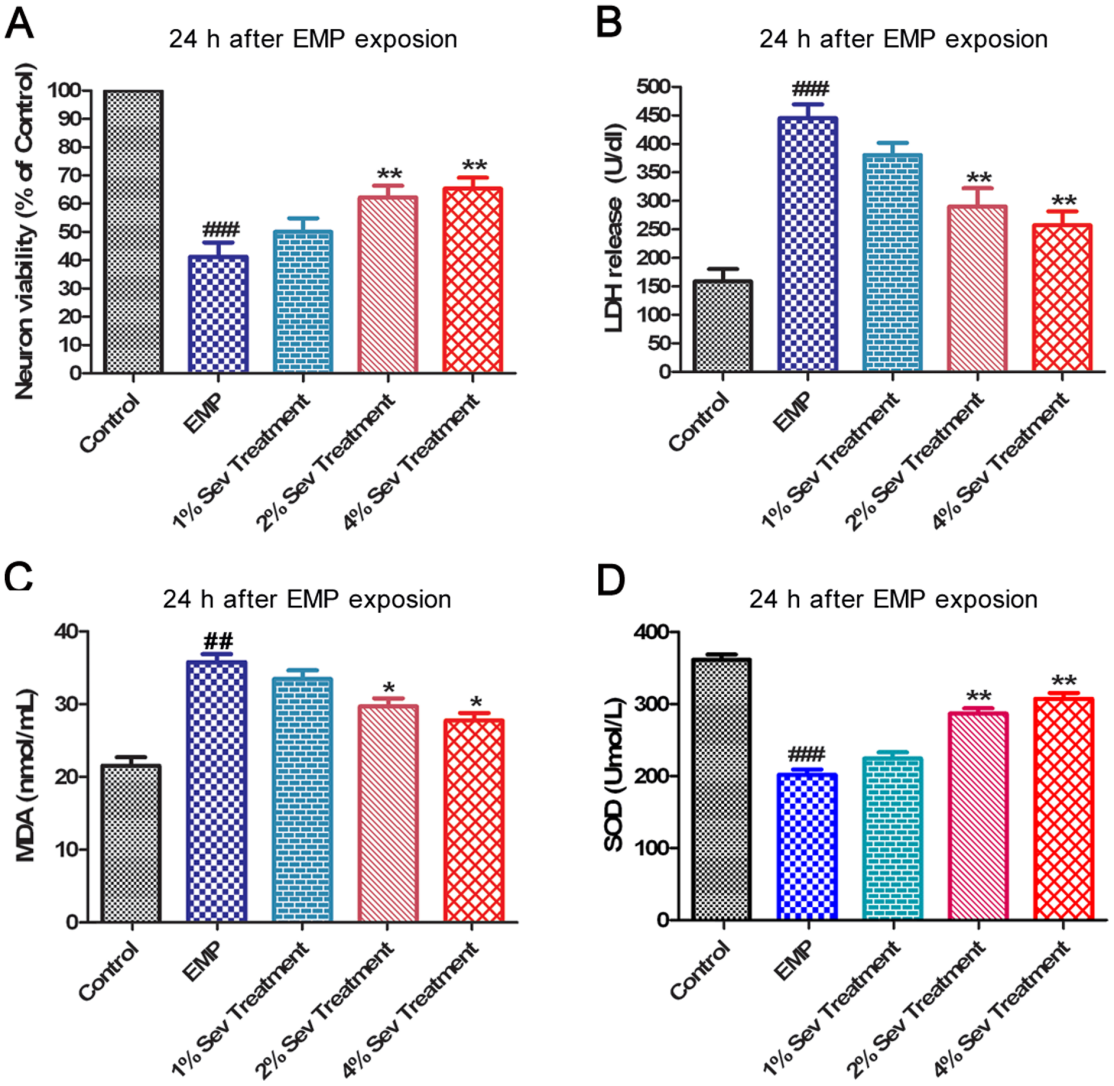
Effect of sevoflurane on cell viability, LDH release, MDA level and SOD activity of cerebral cortical neurons exposed to EMP radiation. The cell viability (**A**), LDH release (**B**), MDA level (**C**) and SOD activity (**D**) were evaluated at 24 h after exposure to EMP radiation. The cell viability in the control group (normal cell group) was normalized to 100%. The values represent the mean (% of control) ± SEM of each group in this experiment. ^##^, *P*<0.01 and ^####^, *P*<0.001 vs. control group; *, *P*<0.05 and **, *P*<0.01 vs. EMP group.

Using cerebral cortical neurons, we investigated the cytoprotective effect of sevoflurane against EMP radiation. Compared with the control group, 24 h after EMP exposure, the cell viability of the EMP group was significantly reduced (*P*<0.01) and LDH release was significantly increased (*P*<0.01). Moreover, 2% and 4% sevoflurane significantly increased cell viability and markedly prevented the elevation of LDH release at 6 or 24 h after EMP exposure. However, there was no significant difference in cell viability and LDH release between the 1% sevoflurane treatment group and the EMP group (Fig. 5A/B)

### 6. Sevoflurane decreased MDA levels and increased SOD activity in cerebral cortical neurons exposed to EMP radiation *in vitro*

MDA is a product of lipid peroxidation and a biomarker for oxidative stress. The MDA content in the EMP group was significantly increased compared with that in the control group at 24 h after EMP exposure (P<0.01). Treatment with 2% or 4% sevoflurane treatment significantly attenuated the elevation of MDA content at 24 h after EMP exposure (P<0.05) (Fig. 5C).

Compared with the control group, a significant reduction in SOD activity was noted 24 h after EMP exposure (*P*<0.001). Treatment with 2% or 4% sevoflurane significantly increased the SOD activity compared with the EMP group at 24 h after EMP exposure (P<0.01) (Fig. 5D). However, 1% sevoflurane treatment did not affect MDA content or SOD activity.

### 7. Sevoflurane alleviated apoptosis of cerebral cortical neurons exposed to EMP radiation *in vitro*

**Figure 6:**
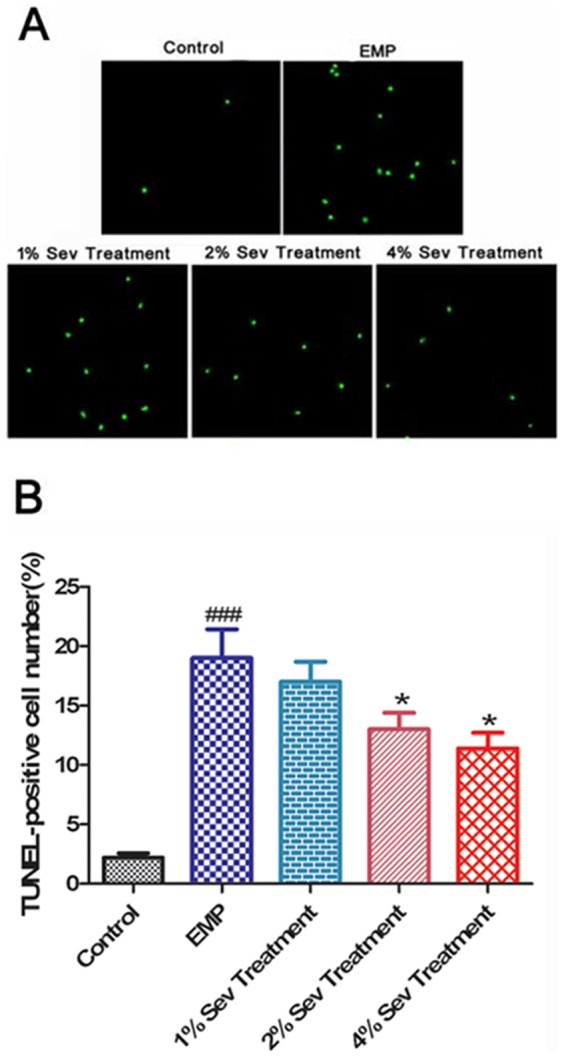
Effect of sevoflurane on apoptosis of cerebral cortical neurons exposed to EMP radiation. **A**, Representative TUNEL staining of cerebral cortical neurons at 24 h after exposure to EMP radiation. The TUNEL-positive neurons are represented in green. Scale bar = 100 mm. **B**, The number of TUNEL-positive cells in each group. ###, *P*<0.001 vs. control group; *, *P*<0.05 vs. EMP group.

TUNEL staining was performed to detect cell apoptosis at 24 h after EMP exposure, and the results are shown in Fig. 6. Compared with the control group, the number of TUNEL-positive cells in the EMP group was markedly increased (*P*<0.001), whereas the number of TUNEL-positive cells in the 2% or 4% sevoflurane groups was significantly less than that in the EMP group (*P*<0.05). However, there was no significant difference in the number of TUNEL-positive cells between the 1% sevoflurane group and the EMP group.

### 8. Sevoflurane regulated the expression of cleaved caspase-3, Bcl-2 and Bax in neurons exposed to EMP radiation *in vitro*

**Figure 7:**
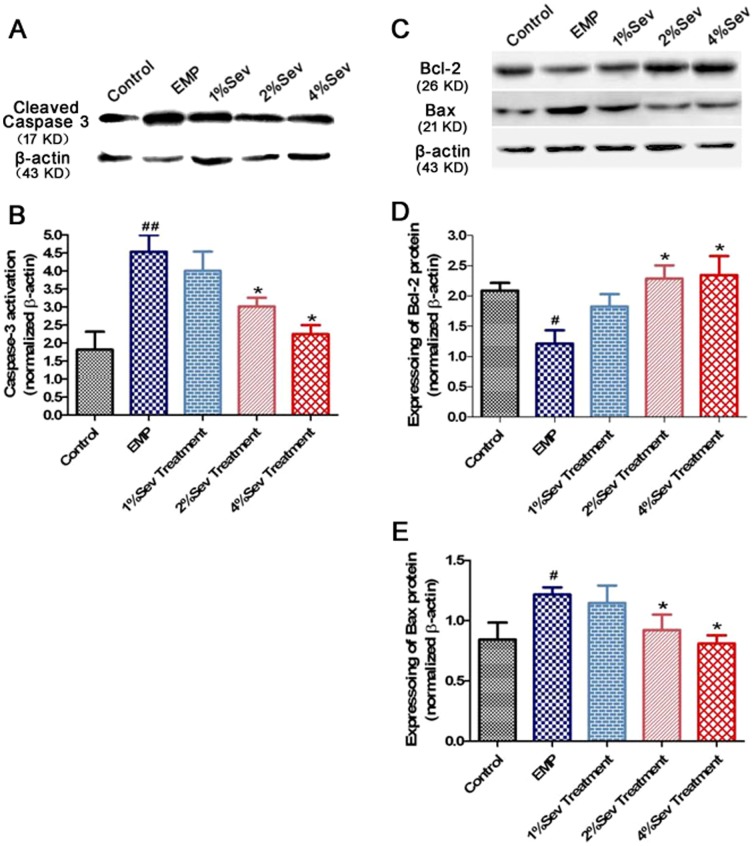
Expression of active caspase-3, Bcl-2 and Bax in cerebral cortical neurons exposed to EMP radiation. **A**, Representative western blot of active caspase-3 at 24 h after exposure to EMP radiation. **B**, Quantification of relative changes in active caspase-3 expression. **C**, Representative western blotting of Bcl-2 and Bax at 24 h after exposure to EMP radiation. **D and E**, Quantification of relative changes in Bcl-2 and Bax expression. The data are normalized to β-actin. ^##^, *P*<0.05 vs. control group; *, *P*<0.05 vs. EMP group.

The expression of cleaved (active) caspase-3 was assessed by western blotting at 24 h after EMP exposure (Figs. 7A and B). Caspase-3 activity was significantly elevated in the EMP group compared with the control group (*P*<0.01). However, 2% or 4% sevoflurane significantly inhibited the expression of cleaved caspase-3 (*P*<0.05).

Extensive evidence suggests that the Bcl-2 family of proteins forms a complex network to regulate apoptosis. Bcl-2 is an anti-apoptotic protein, whereas Bax is pro-apoptotic. As shown in Fig. 7C–E, Bax expression was significantly increased in the EMP group compared with the control group. Treatment with 2% or 4% sevoflurane decreased Bax expression. The Bcl-2 level in the EMP group was significantly decreased compared with that in the control group, whereas 2% or 4% sevoflurane significantly upregulated the expression of Bcl-2 (*P*<0.05). Nevertheless, 1% sevoflurane treatment did not change Caspase-3 activity or the expression of Bcl-2 and Bax compared to EMP group.

## Discussion

The CNS is sensitive to electromagnetic radiation, and pathological damage and neurobehavioral disorders have been observed after EMP exposure [30]. Exposure to electromagnetic fields is a potential health hazard to humans, especially military personnel and/or researchers who work with or can be exposed to this type of electromagnetic field. Therefore, it is of great importance to investigate the biological effects of electromagnetic fields on the CNS and to develop potential preventive strategies.

There is no consensus regarding whether EMP exposure could cause potential detrimental effects in whole animals or isolated cells. Chavdoula et al. reported that Global System for Mobile Telecommunications (GSM)-900 MHz mobile phone electromagnetic radiation caused a large decrease in insect reproductive capacity. This effect was found to be non-thermal and correlated with an increased percentage of induced fragmented DNA and induced cell death in egg chamber cells at early and mid-oogenesis [10]. Although no histopathological changes occurred in rat brains following long-term EMP exposure from GSM-900 mobile phone radiation [31], several studies demonstrated that EMP exposure increased the permeability of the BBB [13] and altered the localization and decreased the levels of tight junction proteins [5]. The negative effect of EMP exposure may depend on the frequency of the electromagnetic field, the type of electrical field applied, the intensity of the power and the thermal or non-thermal effects [32], [33], [34].

In the present study, cerebral cortex neuron injury and neurocognitive impairment were evaluated within 24 h after exposure to EMP radiation. Morphological changes were first observable in cortex neurons at 24 h after EMP exposure. We found many abnormal neurons, and we noted cell bodies that exhibited pyknosis. Furthermore, Nissl staining, FJC staining [35], and neuronal ultrastructures revealed that EMP caused cortical neuron morphological damage. Importantly, learning and memory deficits were also observed after EMP exposure. Therefore, our present study showed that EMP exposure caused acute damage to neurons and short-term neurocognitive impairment. These results differ from our previous study, which demonstrated that EMP exposure could cause long-term neurocognitive impairment in rats at 12 and 18 months after EMP exposure [12].

Sevoflurane is one of the most frequently used volatile general anesthetic agents used during surgical procedures. Sevoflurane is especially useful for pediatric anesthesia because it allows rapid induction and recovery and is less irritating to the airway than other inhaled anesthetics. Although the effects of sevoflurane on neuronal oxidative stress and apoptosis are still controversial, several lines of evidence have suggested that sevoflurane possesses potent neuroprotective effects against oxidative stress injury and apoptosis in the central nervous system. The neuroprotective effects of sevoflurane are related to its ability to decrease the cerebral utilization of oxygen and glucose, inhibit oxidative metabolism in neutrophils [36] and scavenge ROS [37]. Furthermore, sevoflurane shows acute neuroprotective effects by inhibiting lipid peroxidation, lowering MDA levels, and increasing normal pyramidal neuron density in cerebral ischemia reperfusion rats [22]. Another study revealed that exposure with 3.7% sevoflurane for 20 min upregulated the activities of CAT and SOD and induced acute neuroprotection against spinal cord ischemic injury [38]. Meanwhile, sevoflurane alleviated cell apoptosis after brain injury by upregulating Bcl-2 [39]. Wang, J K et al reported that postconditioning with sevoflurane markedly improved spatial learning and memory and reduced apoptotic cell numbers by upregulating Bcl-2 and downregulating Bax, suggesting that the underlying acute protective mechanism of sevoflurane might be linked to reduced apoptosis [40]. Thus, sevoflurane may be an attractive candidate for preventing EMP-induced brain damage.

In this study, rats or cultured cortex neurons were treated with sevoflurane for 20 min after EMP exposure. Our results showed that 2% or 4% sevoflurane reduced neuronal degeneration and alleviated neuronal ultrastructural damage to the mitochondria, rough endoplasmic reticulum and cell nucleus. Interestingly, treatment with sevoflurane improved the cognitive ability of rats exposed to EMP radiation. Several studies have verified that sevoflurane preconditioning protects mitochondria or other organelles from cerebral ischemia/reperfusion injury. Thus, we speculated that the beneficial effects of sevoflurane against EMP exposure may result from the neuroprotective effect of sevoflurane.

We also explored the underlying mechanism of the protective effect of sevoflurane against EMP damage. We demonstrated that at 24 h after EMP exposure, the ultrastructure of cortical neurons changed markedly and was characterized by nuclear membrane folds, collapse and blurred boundaries. All changes in neuronal ultrastructure reflected imbalances in neuronal metabolic functions. Therefore, we speculated that this type of EMP-induced neuronal damage may be related to impaired cell function and metabolic disorders. *In vitro*, we found that EMP exposure reduced SOD activity and increased the MDA level, which was consistent with the previous finding that EMP enhanced oxidative stress [7]. However, sevoflurane increased SOD activity and reduced the MDA level, which implied that sevoflurane exerted a neuroprotective effect against EMP exposure by alleviating oxidative stress. According to a previous study, general anesthetics may abrogate oxidative injury to neurons by preventing the initiation of free radical chain reactions or terminating the propagation of highly reactive radicals [37].

Apoptosis is characterized by specific cell structural changes, including cell shrinkage, nuclear enrichment and DNA breakage [41]. TUNEL staining is a sensitive method for marking early apoptotic cells. In the present study, the number of TUNEL-positive cells increased at 24 h after EMP exposure, indicating that electromagnetic radiation induced DNA damage or apoptotic cell death as reported previously [42], [43]. Caspase-3 can enzymatically digest specific substrates and inhibit DNA repair enzymes, thus destroying cytoskeletal proteins and ribonucleic protein, leading to breakage of the chromosome into small fragments and eventual apoptosis. Moreover, it is known that the pro-apoptotic protein Bax and the anti-apoptotic protein Bcl-2 can migrate from the cytoplasm to mitochondria, which are distributed in a manner that is consistent with mitochondrial release of cytochrome C and caspase [44]. The mitochondrial apoptotic pathway plays an important role in neuronal injury [45], and sevoflurane inhibits cellular apoptosis after brain cerebral ischemic injury by upregulating Bcl-2 expression and reducing Bax expression [39], [40]. Our results showed that sevoflurane reduced caspase-3 and Bax expression and enhanced the expression of the anti-apoptosis protein Bcl-2 after EMP exposure, suggesting that the mitochondrial apoptotic pathway is involved in the protective effects against EMP-induced brain injury. However, the underlying mechanism of the neuroprotective effects of sevoflurane requires further investigation.

In summary, the current study demonstrated that exposure to 400 kV/m EMP induced cerebral cortical neuronal damage and degeneration, apoptosis and cognitive impairment. Sevoflurane conferred protective effects against EMP-induced brain injury by inhibiting neuronal oxidative stress and apoptosis.
